# Electrostatic Self-Assembly of Composite Nanofiber Yarn

**DOI:** 10.3390/polym13010012

**Published:** 2020-12-22

**Authors:** Wei-Chih Wang, Yen-Tse Cheng, Benjamin Estroff

**Affiliations:** 1 Department of Mechanical Engineering, University of Washington, Seattle, WA 98195, USA; birdinhand@gmail.com; 2 Department of Electrical & Computer Engineering, University of Washington, Seattle, WA 98195, USA; 3 Power Mechanical Engineering and National Tsinghua University, Hsinchu City 300, Taiwan; yentse286@hotmail.com; 4 Institute of Nanoengineering and Microsystems, National Tsinghua University, Hsinchu City 300, Taiwan

**Keywords:** fabrics/textiles, polymer fibers, textile composites, conductive nanofiber, electrospinning

## Abstract

Electrospinning polymer fibers is a well-understood process primarily resulting in random mats or single strands. More recent systems and methods have produced nanofiber yarns (NFY) for ease of use in textiles. This paper presents a method of NFY manufacture using a simplified dry electrospinning system to produce self-assembling functional NFY capable of conducting electrical charge. The polymer is a mixture of cellulose nanocrystals (CNC), polyvinyl acrylate (PVA) and poly(3,4-ethylenedioxythiophene) polystyrene sulfonate (PEDOT:PSS). When treated with ethylene glycol (EG) to enhance conductivity, fibers touching the collector plate align to the applied electrostatic field and grow by twisting additional nanofiber polymers injected by the jet into the NFY bundle. The longer the electrospinning continues, the longer and more uniformly twisted the NFY becomes. This process has the added benefit of reducing the electric field required for NFY production from >2.43 kV cm^−1^ to 1.875 kV cm^−1^.

## 1. Introduction

Electronic textiles (also called “e-textiles” or “smart textiles”) refer to electronic systems embedded in clothing. Previously, these specially engineered textiles were designed mainly for extreme weather conditions [1,2,3]. More recently, smart textiles have become more than just body protection. When smart textiles are functionalized, new capabilities become possible, including sensing, signal transmission and reception, and harvesting and storing energy [4,5]. While they are not a replacement for traditional electronics in these roles, smart textiles still present many fascinating and interesting possibilities to enhance fabrics.

To produce electronic textiles, existing spinning systems for yarn fabrication will require modification or replacement. Structurally, yarn is made of a long continuous length of interlocked fibers. Conventional mechanical fiber-spinning technologies cannot produce fiber diameters smaller than a few micrometers, with most current commercial fibers featuring significantly larger diameters for cost efficiency. However, several traditional techniques, such as bicomponent spinning, melt-blowing and flash spinning, have been shown to produce sub-micrometer or nanometer scale fibers in the nonwoven mats [6]. Other methods of fabricating nanofibers, such as phase separation and template synthesis, also show some success in achieving small diameter fibers [7,8]. However, the electrospinning technique is the most versatile, flexible and easiest to use in nanofiber yarn (NFY) production.

Electrospinning has been widely explored to produce fibers with micrometer to nanometer diameters from polymers, composites, ceramics, and metals in the form of solution and melt. Electrospun yarns have been used in membranes, filtration, tissue scaffolds, and wound dressings with more recent applications in the mechanical, electrical, optical, and biomedical fields [9,10]. Previously, electrospun nanofibers were produced in the form of non-woven webs or in random fiber mats. More recently, electrospinning has been able to fabricate twisted yarns for use in weaving, knitting, and embroidery. To generate highly ordered structures such as thread or yarn, current electrospinning systems manipulate the applied electric field and processing parameters or use specialized nanofiber collectors [11,12,13,14]. The collectors can be optimized in architecture, mechanical geometry, dimensions, and dynamic motion to achieve better ordering or length [14,15]. For example, a drum collector can force disordered fibers to be collected in a more highly aligned fashion, or an array of counter-electrodes can align and pattern nanofibers during collection [16,17,18]. Yarn can also be collected by a liquid bath or a rotating a grounded disk coupled to an ungrounded disk to twist fibers into bundles between the parallel electrodes [19,20,21,22,23,24]. Longitudinally uniform and aligned fibers can also be collected by suspending electrospun nanofibers between two grounded ring electrodes, rotating one electrode to twist the nanofibers into yarn [25,26]. However, most of these techniques require extensive modification of current systems or involve complex manipulation [6]. In this article, we present a simple and robust electrospinning method for the self-assembling fabrication of NFY. The innovation lies in the self-triggered thread formation during electrospinning, enabled by the electrostatic forces between conductive nanofibers made from an ethylene glycol (EG)-treated cellulose nanocrystal/polyvinyl alcohol/poly(3,4-ethylenedioxythiophene) polystyrene sulfonate (CNC/PVA/PEDOT:PSS) composite. These forces, in combination with the mechanical whipping motion of nanofibers during electrospinning, assemble highly ordered and oriented threads. This method puts away mechanical components for twisting motion, but triggers the twisting using a current electrospinning system. Furthermore, the introduction of EG enhances the CNC/PVA/PEDOT:PSS composite conductivity, reducing the required applied electric field [27].

## 2. Methodology

**Figure 1:**
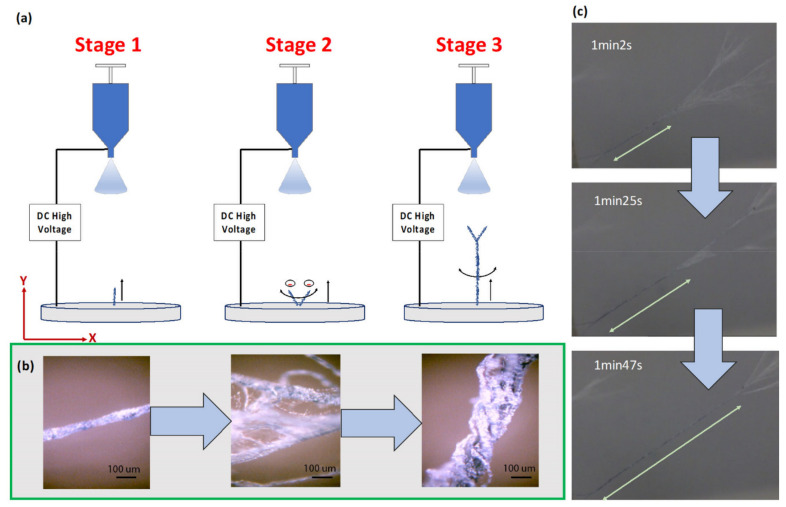
(**a**) Schematic diagram of nanofiber yarn formation. Stage 1: Individual standing fibers populate the collector plate and lengthen. Stage 2: Fiber bundles twist from electrostatic and mechanical forces to form yarn with neighboring fibers. Stage 3: Newly spun nanofibers attach to the growing yarn structure and continue to twist about the longitudinal axis. (**b**) Structural transformation from a single fiber to a yarn, demonstrating collection of single fibers into an organized structure in stage 2 and 3. (Scale bar: 100) (**c**) Time lapse sequence of stage 3 nanofiber yarn formation at 62, 85 and 107 s.

**Figure 2:**
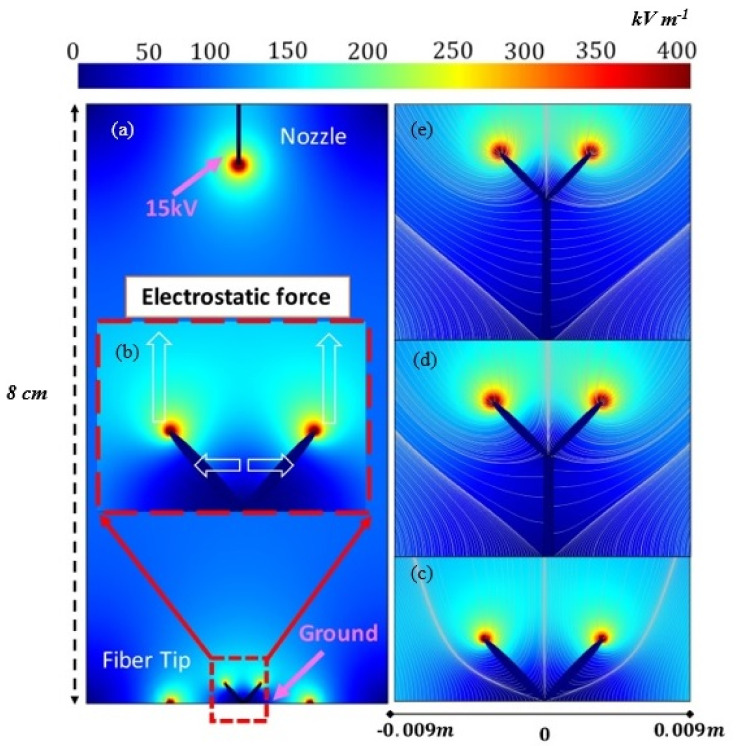
Electric field lines in the region between the needle and the collector. (**a** and **b**) Normalized electric field distribution at stage 2 of fabrication showing model configuration. The black straight line represents the charged tip of the needle. The two ‘rice’ shapes represent the two standing fiber tips attached to the grounded collecting plate. (**c–e**) Electric field lines formed near fiber tips in (**c**) stage 2 and (**d** and **e**) stage 3.

Like conventional electrospinning systems, the proposed method uses electrostatic forces to produce fine nanofibers. This method differs by further using electrostatic repulsion in conductive fibers to align the fibers with the applied field while keeping the tips separated. Combined with the mechanical whipping motion of electrospinning, the process attracts newly spun nanofibers to attach to existing strands, twisting the charged nanofibers into bundles around the longitudinal axis to form the yarn (Figure 1 and Figure 2).

### 2.1. Yarn Formation

The process of yarn formation occurs in three stages. In stage 1, the conductive nanofibers are spun out of the charged droplet of the spinneret by the applied electric field between the spinneret and the collector. The nanofibers break off from the jet and attach to the collector electrode, aligning longitudinally in the direction of electrostatic force, while continuing to swirl from the naturally occurring whipping motion. The conductive properties of the standing fibers result in charge concentrations at their tips, attracting newly-spun fibers from the oppositely-charged jet, lengthening the standing fibers (Figure 1a,b, left image). In stage 2, the increased distance between the fiber tips and the ground plate allows the entire bundle to whip around the anchor point and contact adjacent bundles (Figure 1a). When in contact, the charge concentration provides a transverse repulsion force that keeps the tips separated while the rest of the fiber lengths twist around each other, forming a multi-fiber yarn (Figure 1b). Initially, the repulsive electrostatic force between the tips of plate-attached fibers is dominated by the tip-to-plate field, squeezing the fiber tips together for tighter winding, but as the yarn grows, the impact of the plate-tip field diminishes, resulting in more erratic wrapping (Figure 1b, middle image). In stage 3, the longitudinal electrostatic force from the spinneret begins to dominate, drawing the entire yarn structure into alignment with the applied field (Figure 1a). The fiber tips twist about the longitudinal axis, tightening the wrap as additional nanofiber attaches to the tips (Figure 1b, right image). Figure 1c shows the time-lapsed yarn formation sequence from twisting nanofiber bundles in stage 3. The relative magnitude of the repulsive force between the fiber tips determines the yarn’s wrap tightness at different distances from the ground plate (or electrospun time elapsed). 

### 2.2. Experiment Setup

The setup of the proposed electrospinning system is nearly identical to the conventional configuration, except for the use of a flat metal electrode. In this procedure, a 27-gauge needle is connected to a high-voltage power supply (ES50, Gamma High Voltage Research Inc., Ormond Beach, FL, USA) capable of generating DC voltages up to 50 kV. During testing, the applied DC voltage ranges from 15 to 19 kV depending on the composition of the nanofiber solution. The solution is continuously supplied using a syringe pump (KDS-200, Stoelting, Wood Dale, IL, USA) at a rate of 5 uL min^−1^. Testing was conducted at various distances (7.0 to 8.5 cm) between the spinneret and aluminum foil covered collecting plate. All experiments were conducted at 25 °C room temperature and 70% humidity. 

### 2.3. Finite Element Modelling of Yarn Formation

To explain the electrostatic fiber repelling and twisting motion aided by the electric field, a Finite Element Model (FEM) was created using COMSOL software. The model uses three simple assumptions: First, that a conductive nanofiber that settles on the charged collector plate concentrates charge at the tip as it aligns to the direction of the applied electric field. Second, that fibers spun out of the droplet will have an opposite charge to the collector nanofibers and be attracted to the collector fiber tips. Third, any additional nanofibers that join into a bundle will also concentrate charge at their tips.

The model is based on a basic electrospinning system with one nozzle and a planar collection plate. The nozzle was set to 15 kV and two representative fiber tips (the two ‘black rice’ shapes) were set as the alternate grounds from the ground plate (Figure 2b). To be noted, the use of two tips in simulation is only to demonstrate the physical phenomenon of the electrostatic repulsion and attraction. In practice, there are multiple tips (10 to 20) that are pulled up and spun into multiple bundles. The dimension of the tips and their distance are selected according to observation. The distance between the tip of the needle and ground plate is 8.0 cm. The intensity of the longitudinal and transverse fields at the tips increases as the distance to the needle decreases (Figure 2c–e).

As only the assembly of NFY is of interest, the model only considers stages 2 and 3. In stage 2, the longitudinal electric field concentrates charge at the two fiber tips, attracting (and repelling) the tips vertically while the charge at the tips provides a transverse repulsion force. However, at the start of fiber growth (Figure 2c) the proximity to the ground plate’s field forces the tips closer together; the transverse force is weaker than the longitudinal force due to the lower number of nanofibers involved and the field intensity close to the ground plane. We think that if the distance is sufficiently small, a very high repulsive force break the fibers. However, the nanofibers with align to the longitudinal E force will and form an equilibrium state, helping the charged jet to attract to the fiber tips and impart the whipping motion. From this, the model predicts that fiber bundles near the ground plate will be tightly wound due to the naturally occurring mechanical whipping motion. As the length of the yarn increases (Figure 2c) and more fiber material joins in from the jet, the decreasing distance between the fiber tips and the needle increases the attractive force applied by the longitudinal electric field and the repulsion forces between fibers will dominate. At this stage, the tip repulsion might result in looser wrapping, with the tips at their furthest separation (greatest transverse repulsion force) during the twist. In stage 3, the yarn grows closer to the spinneret (Figure 2d,e) and the attractive longitudinal electric field begins to dominate, bringing the tips closer together as the mechanical swirling motion increases, resulting in tighter winding. 

In summary, while more nanofibers are gradually depositing/growing on the fiber tips, there is a visible variation of the electrostatic field distribution due to the charge concentration at the fiber tips providing a counterforce to the charge of the plate. The growth of the nanofiber yarn isolates the electric fields of the tips from the field from the ground plate (shown in Figure 2). Furthermore, the combination of transverse and longitudinal electrostatic force and the naturally generated mechanical whipping motion of electrospinning [28] are responsible for the overall fiber twisting phenomenon. Eventually, a single standing nanofiber yarn will serve as the main attraction of subsequent nanofibers and turn them into a final yarn.

## 3. Results and Discussion

In the fabrication of the conductive yarn structure, the most important consideration is the electric field that aligns the nanofibers on the collection plate, allowing the twisting behavior. The conductivity of the polymer solution is also critical, as this impacts the induced surface charge for the nanofibers. Our experiments focused on these parameters.

### 3.1. Chemicals and Materials

The three main ingredients are cellulose nanocrystal (CNC), poly(3,4-ethylenedioxythiophene) polystyrene sulfonate (PEDOT:PSS), and polyvinyl alcohol (PVA). The CNC slurry (purchased from the University of Maine) was diluted to 5.7%. The PEDOT:PSS is Clevios™ PH1000 from Heraeus (Leverkusen, Germany). The ethylene glycol (EG) (99.8%) and PVA were purchased from Sigma Aldrich. The PVA and PEDOT:PSS were selected for conductivity and mechanical integrity. Only a viscoelastic material that can undergo strong deformations while being cohesive enough to support the stresses developed during deformation can be drawn into nanofibers. The polymer solution for the spinning experiment was prepared by dissolving the PVA in PEDOT:PSS at 150 °C on a hotplate for 3 h before adding the dimethyl sulfoxide (DMSO) or EG.

**Figure 3:**
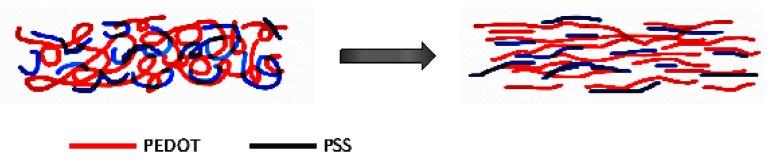
Conformational change of poly(3,4-ethylenedioxythiophene) polystyrene sulfonate (PEDOT:PSS) after solvent (ethylene glycol (EG) or dimethyl sulfoxide (DMSO)) treatment.

The conjugated chain of the π bond on PEDOT offers an electrical path for the transport of electrons, resulting in fascinating optical and electrical properties. Some polar solvents (such as dimethyl sulfoxide (DMSO), EG, and glycerol) have been found to enhance the conductivity of PEDOT:PSS by more than an order of magnitude [12,13,14,29,30,31]. Ouyang et al. indicate a conformational change in the PEDOT chains from a coil structure into a linear structure (Figure 3) is responsible for the increase in conductivity [15,32]. The interaction between the dipoles of the solvent and the dipoles of the PEDOT chains enhances the overall conductivity.

In our experiment, we explored EG and DMSO as polar solvents. Table 1 summarizes the composition of samples used in the experiment. Material A was prepared by dissolving 6% PVA in PEDOT:PSS and mixing with EG or DMSO as a conductive treatment. Material B is 7% PVA in PEDOT:PSS with an additional 2% CNC by weight. The additional CNC added to samples 4 and 5 is mainly to increase mechanical strength. To obtain the required electric field triggering the standing fiber tips, the E field was gradually increased until the tips formed.

### 3.2. Field Considerations

**Figure 4:**
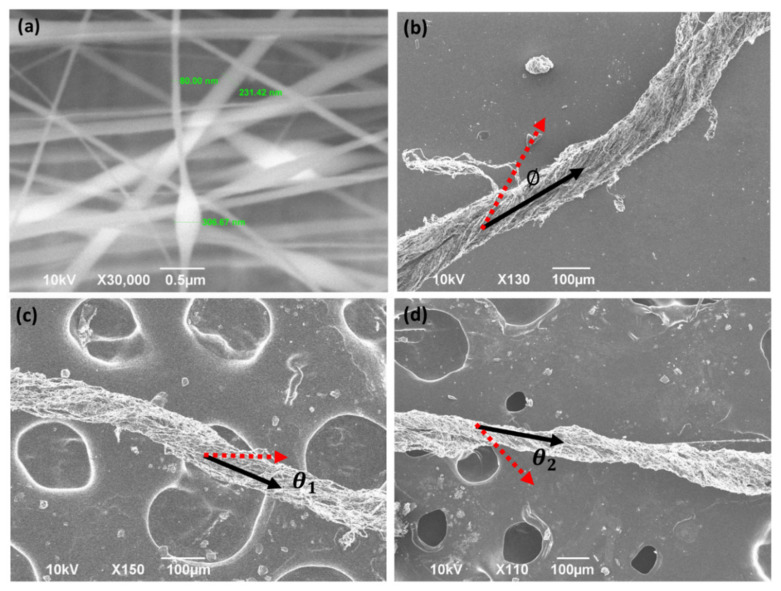
SEM pictures of the PEDOT/PVA structure (**a**) without treatment (**b**) with treatment obtained from the electrostatic fabrication method. (*Ø* = 31°) (**c**) Nanoyarn Material B [see Table 1] after electrospinning for 10 min. (*Ɵ*_1_ = 28°) (**d**) Nanoyarn Material B after electrospinning for 20 min. (*Ɵ*_1_ = 38°).

Different voltages and gap distances were examined for yarn formation. From the test results A, B and C (Table 2), the solvent used in the conductive treatment plays an important role in triggering yarn formation. SEM micrographs of Material A and B with and without solvent treatment are shown in Figure 4. Without solvent, yarn does not form by 2.43 kV cm^−1^. Instead, randomly oriented nanofibers are generated (Figure 4a). For Material A, the minimum E field for triggering the formation of yarn is around 1.875 kV cm^−1^ for either EG or DMSO. From experiments D to J (Table 2), we observed that the addition of EG seems to require a lower E field (>1.76 kV cm^−1^) for yarn formation than DMSO (>2.42 kV cm^−1^). Note that the E field must be below 2.8 kV cm^−1^ to prevent corona discharge between the needle and its surroundings.

### 3.3. Structural Densification-Electrospinning Time

The influence of electrospinning time on yarn density was also investigated. Two runs were conducted using Material B, one electrospun for 10 min and the other for 20 min. The results are shown in Figure 4c,d. Overall, the longer spin time appears to improve the tightness of the yarn wrap, with the 10 min fabrication run producing a roughly 100 µm diameter single yarn (Figure 4c), and the 20 min run producing a 60 µm diameter yarn (Figure 4d). This suggests that longer spin times can densify the yarn structure through twisting larger amounts of the nanofiber into the structure. In addition, longer spin times can not only densify the yarn, but also enable a higher twisting angle of the yarn. In the result of 10 min spin time, the angle of twisting (Ɵ_1_) is around 28°, which can be observed in the SEM picture. This increases to a 38° twisting angle (Ɵ_2_) with 20 min of spinning.

## 4. Conclusions

Electrospinning is a promising method for producing nanofibers on an industrial scale. Fabricating electrospun nanofibers into assembled structures such as yarn increases their utility and creates the potential for new innovative applications of e-textile sensors and systems, such as incorporation with a flexible humidity and airflow sensors [33,34].

Previously proposed methods for assembling nanofibers into yarn included continuous, twisted, and hybrid approaches. However, these tend to involve complicated additions and modifications to existing electrospinning systems. In this paper, we presented a simple yarn fabrication method utilizing conductive polymer materials with no modification to the electrospinning system. We demonstrated the concept of how the yarn forms from nanofiber tips on the ground plate due to the longitudinal and transverse electric fields, and how electrostatic repulsion and mechanical swirling from the electrospinning system form the fibers into bundles using an electrical field FEM. We have successfully demonstrated the concept and conducted a series of studies on how to improve the overall yarn formation by the introduction of a solvent treatment to our base PVA/PEDOT:PSS solution and how different solvents (EG and DMSO) lower the electric field intensity required for yarn formation. We also studied the yarn density as a function of electrospinning time. Additional studies are still required to address some remaining challenges with this technique. We intend to address and optimize the design’s mechanical strength and electrical conductivity for a future publication. 

## 5. Patents

A US patent has been filed prior to the submission of this paper (106F0280-IE(JAP106054-US)).

## Figures and Tables

**Table 1:** Polymer solution composition. Unit: wt.%.

Sample #	Material A (6% PVA Dissolved in PEDOT:PSS)	DMSO	Ethylene Glycol (EG)	CNC
1	93.7%		6.3%	
2	95%	5%		
3	100%			
	Material B (7% PVA dissolved in PEDOT:PSS)			
4	93.1%	4.9%		2%
5	93.1%		4.9%	2%

**Table 2:** Yarn formation due to distance (needle to collector), material composition, and field intensity.

Test #	Voltage (kV)	Distance (cm)	Sample #	E field (kV cm^−1^)	Result
A	15	8.0	1	1.875	Yarn
B	15	8.0	2	1.875	Yarn
C	17	7.0	3	2.43	No yarn
D	17	8.0	4	2.13	No yarn
E	17	7.0	4	2.42	Yarn
F	19	8.5	4	2.24	No yarn
G	19	7.5	4	2.53	Yarn
H	17	8.0	5	2.13	Yarn
I	15	8.0	5	1.87	Yarn
J	15	8.5	5	1.76	Yarn generated after 3 min

## Data Availability

The data presented in this study are available on request from the corresponding author. The data are not publicly available due to patent pending issue.
